# Supplementary material for: Thresholds for post-rebound SHIV control after CCR5 gene-edited autologous hematopoietic cell transplantation
Source: eLife. 2021 Jan 12;10:e57646. doi: 10.7554/eLife.57646 (PMC7803377; doi:10.7554/eLife.57646)
Supplement: Figure 3—source data 1. — We assumed animal weight of 5 Kg. [file elife-57646-fig3-data1.docx]

**Figure 3-source data 1.** Values of the fraction of protected cells in transplant product $f_{p}$, dose or number of HSPCs in transplant product $D$ and time of transplantation $t_{x}$ of each animal for model fitting and projections. We assumed animal weight of 5Kg.

| **Group** | **ID** | $\boldsymbol{\%}\boldsymbol{f}_{\boldsymbol{p}}$ | $\boldsymbol{D}$  **(HSPCs/Kg)** | $\boldsymbol{t}_{\boldsymbol{x}}$  **(weeks after challenge)** |
| --- | --- | --- | --- | --- |
| Control | Z09087 | 0 | 0 | N/A |
|  | Z09106 | 0 | 0 | N/A |
|  | Z09192 | 0 | 0 | N/A |
|  | Z09204 | 0 | 0 | N/A |
|  | A11201 | 0 | 0 | N/A |
| Wild-type transplantation | Z09144 | 0 | 6.45e6 | 55 |
|  | Z08214 | 0 | 4.14e6 | 58 |
|  | A11200 | 0 | 2.08e6 | 56 |
|  | Z09196 | 0 | 2.39e6 | 57 |
|  | Z09125 | 0 | 6.28e6 | 61 |
| ΔCCR5 transplantation | A11219 | 33 | 1.06e7 | 56 |
|  | T10187 | 27 | 6e6 | 57 |
|  | R10159 | 56 | 6.7e6 | 59 |
|  | T10173 | 30 | 1.48e7 | 59 |
|  | Z11151 | 60 | 1.2e7 | 63 |
|  | Z12420 | 54 | 4.82e6 | 63 |
|  | R10155 | 43 | 6e6 | 54 |
|  | Z12216 | 56 | 6.2e6 | 59 |
|  | Z12037 | 30 | 8.25e6 | 58 |
|  | Z12351 | 51 | 2.3e6 | 59 |
|  | Z13133 | 48 | 6e6 | 55 |
|  | Z12417 | 44 | 6e6 | 57 |
