# Supplementary material for: Thresholds for post-rebound SHIV control after CCR5 gene-edited autologous hematopoietic cell transplantation
Source: eLife. 2021 Jan 12;10:e57646. doi: 10.7554/eLife.57646 (PMC7803377; doi:10.7554/eLife.57646)
Supplement: Figure 3—source data 2. — Best fit in bold-red (lowest AIC). The AIC values presented for each statistical assumption is the lowest of 10 runs of the SAEM algorithm with different randomly selected initial guesses. [file elife-57646-fig3-data2.docx]

**Figure 3-source data 2.** Competing models for fitting T cell reconstitution with respective AIC values. Best fit in bold-red (lowest AIC). The AIC values presented for each statistical assumption is the lowest of 10 runs of the SAEM algorithm with different randomly selected initial guesses.

| **Model** | **Mechanistic Assumptions** | **Statistical Assumptions** | **ΔAIC** |
| --- | --- | --- | --- |
| 1 | - Full model as in **eq. 2** in main text. - Does not include compartment *N_p2_* | - All random effects>0. - No correlations among parameters. | 42.4 |
|  |  | - All random effects>0. - $corr(\hat{r}_{s}$,$\hat{r}_{e})\neq0.$ - $corr(N_{0}$,$E_{0})\neq0$. | 24.9 |
|  |  | - All random effects>0. - $corr(\hat{r}_{s}$,$\hat{r}_{e})\neq0.$ - $corr(N_{0}$,$E_{0})\neq0$. - $corr(K_{p}$,$E_{0})\neq0$. - $corr(N_{0}$,$K_{p})\neq0$. | 26.8 |
| 2 | - CD4^+^CCR5^+^ T cells do not downregulate CCR5 ($\lambda_{s}=0$). - Does not include compartment *N_p2_* | - All random effects>0. - No correlations among parameters. | 78.4 |
| 3 | - Thymic export rate of naïve CD4^+^ and CD8^+^ T cells is the same ($\lambda_{e}=\lambda_{f}$). - Does not include compartment *N_p2_* | - All random effects>0. - No correlations among parameters. | 41.7 |
|  |  | - All random effects>0. - $corr(\hat{r}_{s}$,$\hat{r}_{e})\neq0.$ - $corr(N_{0}$,$E_{0})\neq0$. | 25.9 |
|  |  | - All random effects>0. - $corr(\hat{r}_{s}$,$\hat{r}_{e})\neq0.$ - $corr(N_{0}$,$E_{0})\neq0$. - $corr(K_{p}$,$E_{0})\neq0$. - $corr(N_{0}$,$K_{p})\neq0$. | 28.1 |
| 4 | - CD4^+^CCR5^+^ T cells do not downregulate CCR5 ($\lambda_{s}=0$). - Thymic export rate of naïve CD4^+^ and CD8^+^ T cells is the same ($\lambda_{e}=\lambda_{f}$) . - Does not include compartment *N_p2_* | - All random effects>0. - No correlations among parameters. | 69.2 |
| 5 | - CD4^+^CCR5^-^ T cells are long-lived and do not proliferate ($r_{n}=d_{n}=0$). - Does not include compartment *N_p2_* | - All random effects>0. - No correlations among parameters. | 45.9 |
| 6 | - CD4^+^CCR5^-^ T cells are long-lived and do not proliferate ($r_{n}=d_{n}=0$). - CD4^+^CCR5^+^ T cells do not downregulate CCR5 ($\lambda_{s}=0$). - Does not include compartment *N_p2_* | - All random effects>0. - No correlations among parameters. | 70.1 |
| 7 | - CD4^+^CCR5^-^ T cells are long-lived and do not proliferate ($r_{n}=d_{n}=0$). - Thymic export rate of naïve CD4^+^ and CD8^+^ T cells is the same ($\lambda_{e}=\lambda_{f}$). - Does not include compartment *N_p2_* | - All random effects>0. - No correlations among parameters. | 53.5 |
| 8 | - CD4^+^CCR5^-^ T cells are long-lived and do not proliferate ($r_{n}=d_{n}=0$). - Thymic export rate of naïve CD4^+^ and CD8^+^ T cells is the same ($\lambda_{e}=\lambda_{f}$). - CD4^+^CCR5^+^ T cells do not downregulate CCR5 ($\lambda_{s}=0$). - Does not include compartment *N_p2_* | - All random effects>0. - No correlations among parameters. | 91.1 |
| 9 | - CD4^+^CCR5^-^ T cells do not proliferate ($r_{n}=0$). - Does not include compartment *N_p2_* | - All random effects>0. - No correlations among parameters. | 44.5 |
|  |  | - All random effects>0. - $corr(\hat{r}_{s}$,$\hat{r}_{e})\neq0.$ - $corr(N_{0}$,$E_{0})\neq0$. | 27.3 |
|  |  | - All random effects>0. - $corr(\hat{r}_{s}$,$\hat{r}_{e})\neq0.$ - $corr(N_{0}$,$E_{0})\neq0$. - $corr(K_{p}$,$E_{0})\neq0$. - $corr(N_{0}$,$K_{p})\neq0$. | 23.2 |
| 10 | - CD4^+^CCR5^-^ T cells do not proliferate ($r_{n}=0$). - CD4^+^CCR5^+^ T cells do not downregulate CCR5 ($\lambda_{s}=0$). - Does not include compartment *N_p2_* | - All random effects>0. - No correlations among parameters. | 67.0 |
| 11 | - CD4^+^CCR5^-^ T cells do not proliferate ($r_{n}=0$). - Thymic export rate of naïve CD4^+^ and CD8^+^ T cells is the same ($\lambda_{e}=\lambda_{f}$). - Does not include compartment *N_p2_* | - All random effects>0, - no correlations. | 45.7 |
|  |  | - All random effects>0. - $corr(\hat{r}_{s}$,$\hat{r}_{e})\neq0.$ - $corr(N_{0}$,$E_{0})\neq0$. | 21.2 |
|  |  | - All random effects>0. - $corr(\hat{r}_{s}$,$\hat{r}_{e})\neq0.$ - $corr(N_{0}$,$E_{0})\neq0$. - $corr(K_{p}$,$E_{0})\neq0$. - $corr(N_{0}$,$K_{p})\neq0$. | 18.7 |
|  |  | - Random effects equal to zero for parameters: $\hat{r}_{m}$, $\lambda_{n}$, $\lambda_{s}$, $\hat{d}_{n}$, $K_{s}$, and $K_{e}.$ - All random effects>0. - $corr(\hat{r}_{s}$,$\hat{r}_{e})\neq0.$ - $corr(N_{0}$,$E_{0})\neq0$. - $corr(K_{p}$,$E_{0})\neq0$. - $corr(N_{0}$,$K_{p})\neq0$. | 2.8 |
|  |  | - Random effects equal to zero for parameters: $\hat{r}_{m}$, $\lambda_{e}$, $\hat{d}_{n}$, $K_{s}$, and $K_{e}.$ - $corr(\hat{r}_{s}$,$\hat{r}_{e})\neq0.$ - $corr(N_{0}$,$E_{0})\neq0$. - $corr(K_{p}$,$E_{0})\neq0$. - $corr(N_{0}$,$K_{p})\neq0$. | **0.0** |
| 12 | - CD4^+^CCR5^-^ T cells do not proliferate ($r_{n}=0$). - Thymic export rate of naïve CD4^+^ and CD8^+^ T cells is the same ($\lambda_{e}=\lambda_{f}$). - CD4^+^CCR5^+^ T cells do not downregulate CCR5 ($\lambda_{s}=0$). - Does not include compartment *N_p2_* | - All random effects>0. - No correlations among parameters. | 63.8 |
| 13 | - Full model as in **eq. 2** in main text. - Includes compartment *N_p2_* | - All random effects>0. - No correlations among parameters. | 42.2 |
|  |  | - All random effects>0. - $corr(\hat{r}_{s}$,$\hat{r}_{e})\neq0.$ - $corr(N_{0}$,$E_{0})\neq0$. | 26.1 |
|  |  | - All random effects>0. - $corr(\hat{r}_{s}$,$\hat{r}_{e})\neq0.$ - $corr(N_{0}$,$E_{0})\neq0$. - $corr(K_{p}$,$E_{0})\neq0$. - $corr(N_{0}$,$K_{p})\neq0$. | 30.5 |
| 14 | - CD4^+^CCR5^+^ T cells do not downregulate CCR5 ($\lambda_{s}=0$). - Includes compartment *N_p2_* | - All random effects>0. - No correlations among parameters. | 81.3 |
| 15 | - Thymic export rate of naïve CD4^+^ and CD8^+^ T cells is the same ($\lambda_{e}=\lambda_{f}$). - Includes compartment *N_p2_* | - All random effects>0. - No correlations among parameters. | 55.3 |
|  |  | - All random effects>0. - $corr(\hat{r}_{s}$,$\hat{r}_{e})\neq0.$ - $corr(N_{0}$,$E_{0})\neq0$. | 47.1 |
| 16 | - CD4^+^CCR5^+^ T cells do not downregulate CCR5 ($\lambda_{s}=0$). - Thymic export rate of naïve CD4^+^ and CD8^+^ T cells is the same ($\lambda_{e}=\lambda_{f}$) . - Includes compartment *N_p2_* | - All random effects>0. - No correlations among parameters. | 71.7 |
| 17 | - CD4^+^CCR5^-^ T cells are long-lived and do not proliferate ($r_{n}=d_{n}=0$). - Includes compartment *N_p2_* | - All random effects>0. - No correlations among parameters. | 44.5 |
| 18 | - CD4^+^CCR5^-^ T cells are long-lived and do not proliferate ($r_{n}=d_{n}=0$). - CD4^+^CCR5^+^ T cells do not downregulate CCR5 ($\lambda_{s}=0$). - Includes compartment *N_p2_* | - All random effects>0. - No correlations among parameters. | 67.3 |
| 19 | - CD4^+^CCR5^-^ T cells are long-lived and do not proliferate ($r_{n}=d_{n}=0$). - Thymic export rate of naïve CD4^+^ and CD8^+^ T cells is the same ($\lambda_{e}=\lambda_{f}$). - Includes compartment *N_p2_* | - All random effects>0. - No correlations among parameters. | 52.6 |
| 20 | - CD4^+^CCR5^-^ T cells are long-lived and do not proliferate ($r_{n}=d_{n}=0$). - Thymic export rate of naïve CD4^+^ and CD8^+^ T cells is the same ($\lambda_{e}=\lambda_{f}$). - CD4^+^CCR5^+^ T cells do not downregulate CCR5 ($\lambda_{s}=0$). - Includes compartment *N_p2_* | - All random effects>0. - No correlations among parameters. | 87.4 |
| 21 | - CD4^+^CCR5^-^ T cells do not proliferate ($r_{n}=0$). - Includes compartment *N_p2_* | - All random effects>0. - No correlations among parameters. | 44.4 |
|  |  | - All random effects>0. - $corr(\hat{r}_{s}$,$\hat{r}_{e})\neq0.$ - $corr(N_{0}$,$E_{0})\neq0$. | 18.6 |
|  |  | - All random effects>0. - $corr(\hat{r}_{s}$,$\hat{r}_{e})\neq0.$ - $corr(N_{0}$,$E_{0})\neq0$. - $corr(K_{p}$,$E_{0})\neq0$. - $corr(N_{0}$,$K_{p})\neq0$. | 15.0 |
| 22 | - CD4^+^CCR5^-^ T cells do not proliferate ($r_{n}=0$). - CD4^+^CCR5^+^ T cells do not downregulate CCR5 ($\lambda_{s}=0$). - Includes compartment *N_p2_* | - All random effects>0. - No correlations among parameters. | 68.0 |
| 23 | - CD4^+^CCR5^-^ T cells do not proliferate ($r_{n}=0$). - Thymic export rate of naïve CD4^+^ and CD8^+^ T cells is the same ($\lambda_{e}=\lambda_{f}$). - Includes compartment *N_p2_* | - All random effects>0, - no correlations. | 44.3 |
|  |  | - All random effects>0. - $corr(\hat{r}_{s}$,$\hat{r}_{e})\neq0.$ - $corr(N_{0}$,$E_{0})\neq0$. | 21.3 |
|  |  | - All random effects>0. - $corr(\hat{r}_{s}$,$\hat{r}_{e})\neq0.$ - $corr(N_{0}$,$E_{0})\neq0$. - $corr(K_{p}$,$E_{0})\neq0$. - $corr(N_{0}$,$K_{p})\neq0$. | 18.5 |
|  |  | - Random effects equal to zero for parameters: $\hat{r}_{m}$, $\lambda_{n}$, $\lambda_{s}$, $\hat{d}_{n}$, $K_{s}$, and $K_{e}.$ - All random effects>0. - $corr(\hat{r}_{s}$,$\hat{r}_{e})\neq0.$ - $corr(N_{0}$,$E_{0})\neq0$. - $corr(K_{p}$,$E_{0})\neq0$. - $corr(N_{0}$,$K_{p})\neq0$. | 2.2 |
|  |  | - Random effects equal to zero for parameters: $\hat{r}_{m}$, $\lambda_{e}$, $\hat{d}_{n}$, $K_{s}$, and $K_{e}.$ - $corr(\hat{r}_{s}$,$\hat{r}_{e})\neq0.$ - $corr(N_{0}$,$E_{0})\neq0$. - $corr(K_{p}$,$E_{0})\neq0$. - $corr(N_{0}$,$K_{p})\neq0$. | **0.2** |
| 24 | - CD4^+^CCR5^-^ T cells do not proliferate ($r_{n}=0$). - Thymic export rate of naïve CD4^+^ and CD8^+^ T cells is the same ($\lambda_{e}=\lambda_{f}$). - CD4^+^CCR5^+^ T cells do not downregulate CCR5 ($\lambda_{s}=0$). - Includes compartment *N_p2_* | - All random effects>0. - No correlations among parameters. | 64.2 |
