# Supplementary material for: Thresholds for post-rebound SHIV control after CCR5 gene-edited autologous hematopoietic cell transplantation
Source: eLife. 2021 Jan 12;10:e57646. doi: 10.7554/eLife.57646 (PMC7803377; doi:10.7554/eLife.57646)
Supplement: Figure 3—source data 3. — RSE: relative standard error. Empty fields represent a standard deviation of random effects, σψ, fixed to zero. Values of ψ¯ for Kp,N(t0),S(t0),M(t0), and E(t0) shown here are in log10 cell counts/μL assuming a blood volume of of 3 × 105 μL (calculated assuming blood:weight ratio of 60 mL/kg and body weight of 5 kg). Red values represent an RSE greater than 100% implying that the number of data points may not be enough to estimate the respective parameter. [file elife-57646-fig3-data3.docx]

**Figure 3-source data 3.** Population parameter estimates for the best fits of the model in **equation 2** in the main text (lowest AIC in **Figure 3-source data 2**) to the T cell reconstitution dynamics. RSE: relative standard error. Empty fields represent a standard deviation of random effects, $\sigma_{\psi}$, fixed to zero. Values of $\bar{\psi}$ for $K_{p},N\left( t_{0} \right),S\left( t_{0} \right),M\left( t_{0} \right),$ and $E\left( t_{0} \right)$ shown here are in log_10_ cell counts/ μL assuming a blood volume of of 3×10^5^ μL (calculated assuming blood:weight ratio of 60mL/Kg and body weight of 5Kg). Red values represent an RSE greater than 100% implying that the number of data points may not be enough to estimate the respective parameter.

| **Parameter** | $\bar{\boldsymbol{\psi}}$ | $\boldsymbol{\sigma}_{\boldsymbol{\psi}}$ | **%RSE for:** | |
| --- | --- | --- | --- | --- |
|  |  |  | $\bar{\boldsymbol{\psi}}$ | $\boldsymbol{\sigma}_{\boldsymbol{\psi}}$ |
| ${\hat{\boldsymbol{r}}}_{\boldsymbol{p}}$ | 0.05 (0.04) | 0.39 (0.47) | 20 (21) | 23 (23) |
| ${\hat{\boldsymbol{r}}}_{\boldsymbol{s}}$ | 0.11 (0.1) | 0.41 (0.39) | 10 (10) | 18 (19) |
| ${\hat{\boldsymbol{r}}}_{\boldsymbol{m}}$ | 0.03 (0.03) |  | 51 (55) |  |
| ${\hat{\boldsymbol{r}}}_{\boldsymbol{e}}$ | 0.08 (0.07) | 0.56 (0.49) | 15 (14) | 18 (20) |
| ${\hat{\boldsymbol{d}}}_{\boldsymbol{n}}$ | 8.2 (5.38) |  | 36 (37) |  |
| $\boldsymbol{\lambda}_{\boldsymbol{e}}\boldsymbol{=}\boldsymbol{\lambda}_{\boldsymbol{f}}$ | 0.003 (0.004) |  | 43 (39) |  |
| $\boldsymbol{\lambda}_{\boldsymbol{s}}$ | 0.014 (0.015) | 0.2 (0.32) | 24 (25) | 130 (59) |
| $\boldsymbol{\lambda}_{\boldsymbol{n}}$ | 0.003 (0.004) | 0.27 (0.27) | 27 (24) | 40 (42) |
| $\boldsymbol{\lambda}_{\boldsymbol{m}}$ | 0.07 (0.08) |  | 27 (24) |  |
| $\boldsymbol{K}_{\boldsymbol{p}}$ | 3.2 (3.2) | 0.23 (0.23) | 1 (1) | 16 (16) |
| $\boldsymbol{K}_{\boldsymbol{s}}$ | 0.13 (0.18) |  | 21 (19) |  |
| $\boldsymbol{K}_{\boldsymbol{m}}$ | 0.75 (0.77) | 0.28 (0.29) | 28 (29) | 22 (21) |
| $\boldsymbol{K}_{\boldsymbol{e}}$ | 0.15 (0.2) |  | 19 (17) |  |
| $\boldsymbol{N(}\boldsymbol{t}_{\boldsymbol{0}}\boldsymbol{)}$ | 1.9 (1.8) | 0.13 (0.15) | 1 (1) | 27 (24) |
| $\boldsymbol{S(}\boldsymbol{t}_{\boldsymbol{0}}\boldsymbol{)}$ | 0.64 (0.64) | 0.27 (0.28) | 1 (1) | 23 (23) |
| $\boldsymbol{M(}\boldsymbol{t}_{\boldsymbol{0}}\boldsymbol{)}$ | 1.0 (1.0) | 0.14 (0.18) | 2 (2) | 73 (58) |
| $\boldsymbol{E(}\boldsymbol{t}_{\boldsymbol{0}}\boldsymbol{)}$ | 1.3 (1.4) | 0.4 (0.34) | 2 (1) | 21 (22) |
|  | **Parameter value** | | **%RSE** | |
| $\boldsymbol{corr(}{\hat{\boldsymbol{r}}}_{\boldsymbol{s}}$**,**${\hat{\boldsymbol{r}}}_{\boldsymbol{e}}\boldsymbol{)}$ | 0.87 (0.82) | | 9 (13) | |
| $\boldsymbol{corr}\boldsymbol{(}\boldsymbol{N}_{\boldsymbol{0}}$**,**$\boldsymbol{E}_{\boldsymbol{0}}\boldsymbol{)}$ | 0.99 (0.99) | | 14 (12) | |
| $\boldsymbol{corr}\boldsymbol{(}\boldsymbol{K}_{\boldsymbol{p}}$**,**$\boldsymbol{E}_{\boldsymbol{0}}\boldsymbol{)}$ | 0.8 (0.73) | | 15.5 (22) | |
| $\boldsymbol{corr}\boldsymbol{(}\boldsymbol{N}_{\boldsymbol{0}}$**,**$\boldsymbol{K}_{\boldsymbol{p}}\boldsymbol{)}$ | 0.74 (0.6) | | 26 (35) | |
| $\boldsymbol{\sigma}_{\boldsymbol{N}}$ | 0.2 (0.2) | | 4 (4) | |
| $\boldsymbol{\sigma}_{\boldsymbol{S}}$ | 0.16 (0.16) | | 4 (4) | |
| $\boldsymbol{\sigma}_{\boldsymbol{C}}$ | 0.19 (0.19) | | 4 (4) | |
| $\boldsymbol{\sigma}_{\boldsymbol{E}}$ | 0.18 (0.19) | | 11 (11) | |
| $\boldsymbol{\sigma}_{\boldsymbol{M}}$ | 0.21 (0.21) | | 12 (12) | |
