# Supplementary material for: Thresholds for post-rebound SHIV control after CCR5 gene-edited autologous hematopoietic cell transplantation
Source: eLife. 2021 Jan 12;10:e57646. doi: 10.7554/eLife.57646 (PMC7803377; doi:10.7554/eLife.57646)
Supplement: Figure 3—source data 4. — Values obtained for N(t0),S(t0),M(t0), and E(t0) shown here are in log10 cell counts/μL assuming a blood volume of of 3 × 105 μL (calculated assuming blood:weight ratio of 60 mL/kg and body weight of 5 kg). Initial values for the control group where obtained assuming steady state. [file elife-57646-fig3-data4.docx]

**Figure 3-source data 4.** Individual parameter estimates for the best fits of the model in **equation 2** in the main text (lowest AIC in **Figure 3-source data 2**) to the T cell reconstitution dynamics. Values obtained for $N\left( t_{0} \right),S\left( t_{0} \right),M\left( t_{0} \right),$ and $E\left( t_{0} \right)$ shown here are in log_10_ cell counts/ μL assuming a blood volume of of 3×10^5^ μL (calculated assuming blood:weight ratio of 60mL/Kg and body weight of 5Kg). Initial values for the control group where obtained assuming steady state.

|  | **Control** | | | | | **WT-Transplant** | | | | | **ΔCCR5-Transplant** | | | | | | | | | | | | |
| --- | --- | --- | --- | --- | --- | --- | --- | --- | --- | --- | --- | --- | --- | --- | --- | --- | --- | --- | --- | --- | --- | --- | --- |
| **Par.**  **ID** | **Z09087** | **Z09106** | **Z09192** | **Z09204** | **A11201** | **Z09144** | **Z08214** | **A11200** | **Z09196** | **Z09125** | **A11219** | **T10187** | **R10159** | **T10173** | **Z11151** | **Z12420** | **R10155** | **Z12216** | **Z12037** | **Z12351** | **Z13133** | **Z12417** |  |
| ${\hat{\boldsymbol{r}}}_{\boldsymbol{p}}^{\boldsymbol{j}}$  **(1/day)** | 0.05 | 0.05 | 0.05 | 0.05 | 0.05 | 0.06 | 0.04 | 0.04 | 0.07 | 0.07 | 0.03 | 0.05 | 0.04 | 0.04 | 0.05 | 0.08 | 0.05 | 0.09 | 0.05 | 0.05 | 0.04 | 0.08 |  |
| ${\hat{\boldsymbol{r}}}_{\boldsymbol{s}}^{\boldsymbol{j}}$  **(1/day)** | 0.10 | 0.10 | 0.10 | 0.07 | 0.11 | 0.12 | 0.14 | 0.07 | 0.20 | 0.07 | 0.12 | 0.12 | 0.14 | 0.19 | 0.07 | 0.09 | 0.09 | 0.08 | 0.22 | 0.06 | 0.14 | 0.14 |  |
| ${\hat{\boldsymbol{r}}}_{\boldsymbol{m}}^{\boldsymbol{j}}$ **(1/day)** | 0.03 | 0.03 | 0.03 | 0.03 | 0.03 | 0.03 | 0.03 | 0.03 | 0.03 | 0.03 | 0.03 | 0.03 | 0.03 | 0.03 | 0.03 | 0.03 | 0.03 | 0.03 | 0.03 | 0.03 | 0.03 | 0.03 |  |
| ${\hat{\boldsymbol{r}}}_{\boldsymbol{e}}^{\boldsymbol{j}}$  **(1/day)** | 0.05 | 0.07 | 0.07 | 0.04 | 0.07 | 0.10 | 0.10 | 0.06 | 0.19 | 0.04 | 0.07 | 0.11 | 0.11 | 0.15 | 0.05 | 0.10 | 0.05 | 0.05 | 0.17 | 0.03 | 0.13 | 0.15 |  |
| ${\hat{\boldsymbol{d}}}_{\boldsymbol{n}}^{\boldsymbol{j}}$ **(1/day)** | 0.02 | 0.04 | 0.03 | 0.03 | 0.03 | 0.02 | 0.03 | 0.03 | 0.02 | 0.02 | 0.04 | 0.03 | 0.03 | 0.03 | 0.03 | 0.03 | 0.03 | 0.02 | 0.03 | 0.03 | 0.04 | 0.03 |  |
| $\boldsymbol{\lambda}_{\boldsymbol{e}}^{\boldsymbol{j}}$  **(1/day)** | 0.003 | 0.003 | 0.003 | 0.003 | 0.003 | 0.003 | 0.003 | 0.003 | 0.003 | 0.003 | 0.003 | 0.003 | 0.003 | 0.003 | 0.003 | 0.003 | 0.003 | 0.003 | 0.003 | 0.003 | 0.003 | 0.003 |  |
| $\boldsymbol{\lambda}_{\boldsymbol{n}}^{\boldsymbol{j}}$  **(1/day)** | 0.002 | 0.004 | 0.003 | 0.003 | 0.003 | 0.003 | 0.003 | 0.003 | 0.002 | 0.003 | 0.004 | 0.003 | 0.003 | 0.003 | 0.003 | 0.004 | 0.004 | 0.002 | 0.003 | 0.003 | 0.004 | 0.003 |  |
| $\boldsymbol{\lambda}_{\boldsymbol{s}}^{\boldsymbol{j}}$  **(1/day)** | 0.01 | 0.01 | 0.01 | 0.01 | 0.01 | 0.02 | 0.01 | 0.01 | 0.02 | 0.01 | 0.01 | 0.01 | 0.01 | 0.01 | 0.01 | 0.01 | 0.02 | 0.01 | 0.02 | 0.01 | 0.01 | 0.01 |  |
| $\boldsymbol{\lambda}_{\boldsymbol{m}}^{\boldsymbol{j}}$ **(1/day)** | 0.07 | 0.07 | 0.07 | 0.07 | 0.07 | 0.07 | 0.07 | 0.07 | 0.07 | 0.07 | 0.07 | 0.07 | 0.07 | 0.07 | 0.07 | 0.07 | 0.07 | 0.07 | 0.07 | 0.07 | 0.07 | 0.07 |  |
| $\boldsymbol{K}_{\boldsymbol{p}}^{\boldsymbol{j}}$  $\left( \boldsymbol{cells} \right)$ | 10^8.9^ | 10^8.8^ | 10^9.0^ | 10^8.8^ | 10^8.7^ | 10^8.6^ | 10^8.7^ | 10^8.7^ | 10^8.5^ | 10^8.7^ | 10^8.5^ | 10^8.7^ | 10^8.8^ | 10^8.5^ | 10^8.8^ | 10^8.5^ | 10^8.9^ | 10^8.8^ | 10^8.0^ | 10^8.4^ | 10^8.2^ | 10^8.7^ |  |
| $\boldsymbol{K}_{\boldsymbol{s}}^{\boldsymbol{j}}$ $\left( \frac{\boldsymbol{cells}}{\boldsymbol{\mu L}} \right)$ | 2134 | 1535 | 2350 | 1625 | 1274 | 923 | 1214 | 1173 | 739 | 1359 | 735 | 1117 | 1425 | 766 | 1636 | 779 | 1790 | 1654 | 275 | 650 | 407 | 1124 |  |
| $\boldsymbol{K}_{\boldsymbol{m}}^{\boldsymbol{j}}$ $\left( \frac{\boldsymbol{cells}}{\boldsymbol{\mu L}} \right)$ | 678 | 718 | 862 | 277 | 439 | 56 | 398 | 315 | 233 | 307 | 112 | 225 | 327 | 113 | 289 | 154 | 404 | 367 | 52 | 126 | 112 | 281 |  |
| $\boldsymbol{K}_{\boldsymbol{e}}^{\boldsymbol{j}}$ $\left( \frac{\boldsymbol{cells}}{\boldsymbol{\mu L}} \right)$ | 2033 | 1462 | 2239 | 1548 | 1214 | 880 | 1156 | 1117 | 704 | 1294 | 700 | 1065 | 1358 | 730 | 1559 | 742 | 1706 | 1576 | 262 | 620 | 388 | 1071 |  |
| $\boldsymbol{N}^{\boldsymbol{j}}\left( \boldsymbol{t}_{\boldsymbol{0}} \right) \left( \frac{\boldsymbol{cells}}{\boldsymbol{\mu L}} \right)$ | 1452 | 621 | 1350 | 1253 | 832 | 65.6 | 58.6 | 94.6 | 54.5 | 83.4 | 58.2 | 76.3 | 80.1 | 80.0 | 89.0 | 53.4 | 81.8 | 116.9 | 33.9 | 54.8 | 56.8 | 61.9 |  |
| $\boldsymbol{S}^{\boldsymbol{j}}\left( \boldsymbol{t}_{\boldsymbol{0}} \right) \left( \frac{\boldsymbol{cells}}{\boldsymbol{\mu L}} \right)$ | 109 | 82 | 135 | 164 | 69 | 2.7 | 1.9 | 8.3 | 1.6 | 2.7 | 3.5 | 6.8 | 7.4 | 8.5 | 6.3 | 4.5 | 3.6 | 10.2 | 2.2 | 6.3 | 4.3 | 3.5 |  |
| $\boldsymbol{M}^{\boldsymbol{j}}\boldsymbol{(}\boldsymbol{t}_{\boldsymbol{0}}\boldsymbol{)}$ $\left( \frac{\boldsymbol{cells}}{\boldsymbol{\mu L}} \right)$ | 320 | 412 | 496 | 155 | 258 | 8.9 | 9.6 | 14.4 | 11.1 | 10.4 | 11.0 | 10.6 | 10.8 | 10.9 | 11.8 | 11.9 | 11.0 | 10.7 | 8.8 | 9.2 | 13.8 | 9.1 |  |
| $\boldsymbol{E}^{\boldsymbol{j}}\boldsymbol{(}\boldsymbol{t}_{\boldsymbol{0}}\boldsymbol{)}$ $\left( \frac{\boldsymbol{cells}}{\boldsymbol{\mu L}} \right)$ | 1000 | 957 | 1191 | 621 | 561 | 16.3 | 12.7 | 48.4 | 9.1 | 35.3 | 11.0 | 26.1 | 31.9 | 27.1 | 44.3 | 8.7 | 35.8 | 95.6 | 1.9 | 9.0 | 8.8 | 14.5 |  |
