# Supplementary material for: Thresholds for post-rebound SHIV control after CCR5 gene-edited autologous hematopoietic cell transplantation
Source: eLife. 2021 Jan 12;10:e57646. doi: 10.7554/eLife.57646 (PMC7803377; doi:10.7554/eLife.57646)
Supplement: Figure 5—source data 1. — Best fit in bold-red (lowest AIC). [file elife-57646-fig5-data1.docx]

**Figure 5-source data 1.** Competing models for fitting T cell and viral dynamics (**equations 2-3** in main text) using the best model in **Figure 3-source data 2** and fixing parameter values as in **Figure 3-source data 3**, with AIC values. Best fit in bold-red (lowest AIC).

| **Model** | **Mechanistic Assumptions** | **Statistical Assumptions** | **ΔAIC** |
| --- | --- | --- | --- |
| 1 | - SHIV-specific CD8^+^ T cells reduce virus production only ($\theta>0, \kappa=0$). - Immunity is lost during TBI: ($\omega_{8}$, $I_{50}$ and $d_{h}$ different during acute infection and after ATI). - SHIV-infection enhances activation of CD4^+^CCR5^-^ T cells leading to replenishment of CD4^+^CCR5^+^ T cells, and transient reduction of the CD4^+^CCR5^-^ compartment after ATI ($\omega_{4}>0$). - Does not include compartment *N_p2_* | - $\sigma_{t_{sa}}=1, \sigma_{\pi}=0.5$. - ${\psi_{j}^{ATI}=10}^{{\bar{\psi}+n}_{j}+\varsigma_{\psi,\mathrm{ATI}}}$ for $\omega_{8}$and $I_{50}$, and $\psi_{j}^{ATI}=\bar{\psi}e^{n_{j}+\varsigma_{\psi,\mathrm{ATI}}}$ for $d_{h}$. - $\theta=1/\mu L$. - $corr(I_{50}$,$d_{h})\neq0.$ - $corr(\omega_{8}$,$d_{h})\neq0$. - $corr(I_{50},\omega_{8})\neq0$. - $corr(\pi$,$\beta)\neq0$. | 188.9 |
|  |  | - $\sigma_{t_{sa}}=1, \sigma_{\pi}=0.5$. - ${\psi_{j}^{ATI}=10}^{{\bar{\psi}+n}_{j}+\varsigma_{\psi,\mathrm{ATI}}}$ for $\omega_{8}$and $I_{50}$, and $\psi_{j}^{ATI}=\bar{\psi}e^{n_{j}+\varsigma_{\psi,\mathrm{ATI}}}$ for $d_{h}$. - $\theta=1/\mu L$. - $corr(\hat{r}_{s},\lambda_{n})\neq0.$ - $corr(\hat{r}_{e},\lambda_{n})\neq0.$ - $corr(I_{50}$,$d_{h})\neq0.$ - $corr(\omega_{8}$,$d_{h})\neq0$. - $corr(I_{50},\omega_{8})\neq0$. - $corr(\pi$,$\beta)\neq0$. - $corr(k_{t}$,$k_{h})\neq0.$ - $corr(\pi$,$d_{h})\neq0$. - $corr(I_{50},\beta)\neq0$. - $corr(\pi$,$I_{50})\neq0$. - $corr(\omega_{8}$,$\beta)\neq0$. - $corr(\pi$,$\omega_{8})\neq0$. - $t_{sa}^{j}$ for ΔCCR5 and transplant groups was modeled as $\psi_{j}=\bar{\psi}e^{n_{j}+\varsigma_{t_{sa},\Delta CCR5}}$ and $\psi_{j}=\bar{\psi}e^{n_{j}+\varsigma_{t_{sa},\mathrm{WT}}}$, respectively. - $K_{p}^{j}$ for ΔCCR5 and transplant groups was modeled as $\psi_{j}={10}^{{\bar{\psi}+n}_{j}+\varsigma_{K_{p},\Delta CCR5}}$ and $\psi_{j}={10}^{{\bar{\psi}+n}_{j}+\varsigma_{K_{p},WT}}$ respectively. | 105.6 |
|  |  | - $\sigma_{t_{sa}}=1, \sigma_{\pi}=0.5$. - ${\psi_{j}^{ATI}=10}^{{\bar{\psi}+n}_{j}+\varsigma_{\psi,\mathrm{ATI}}}$ for $\omega_{8}$and $I_{50}$, and $\psi_{j}^{ATI}=\bar{\psi}e^{n_{j}+\varsigma_{\psi,\mathrm{ATI}}}$ for $d_{h}$. - $\theta=1/\mu L$. - $corr(\hat{r}_{s},\lambda_{n})\neq0.$ - $corr(\hat{r}_{e},\lambda_{n})\neq0.$ - $corr(\hat{r}_{s},\beta)\neq0.$ - $corr(\hat{r}_{e},\beta)\neq0.$ - $corr(\hat{r}_{s},\pi)\neq0.$ - $corr(\hat{r}_{e},\pi)\neq0.$ - $corr(\beta,\lambda_{n})\neq0.$ - $corr(\pi,\lambda_{n})\neq0.$ - $corr(I_{50}$,$d_{h})\neq0.$ - $corr(\omega_{8}$,$d_{h})\neq0$. - $corr(I_{50},\omega_{8})\neq0$. - $corr(\pi$,$\beta)\neq0$. - $corr(k_{t}$,$k_{h})\neq0.$ - $t_{sa}^{j}$ for ΔCCR5 and transplant groups was modeled as $\psi_{j}=\bar{\psi}e^{n_{j}+\varsigma_{t_{sa},\Delta CCR5}}$ and $\psi_{j}=\bar{\psi}e^{n_{j}+\varsigma_{t_{sa},\mathrm{WT}}}$, respectively. - $K_{p}^{j}$ for ΔCCR5 and transplant groups was modeled as $\psi_{j}={10}^{{\bar{\psi}+n}_{j}+\varsigma_{K_{p},\Delta CCR5}}$ and $\psi_{j}={10}^{{\bar{\psi}+n}_{j}+\varsigma_{K_{p},WT}}$ respectively. | 163.7 |
|  |  | - $\sigma_{t_{sa}}=1, \sigma_{\pi}=0.5$. - ${\psi_{j}^{ATI}=10}^{{\bar{\psi}+n}_{j}+\varsigma_{\psi,\mathrm{ATI}}}$ for $\omega_{8}$and $I_{50}$, and $\psi_{j}^{ATI}=\bar{\psi}e^{n_{j}+\varsigma_{\psi,\mathrm{ATI}}}$ for $d_{h}$. - $\theta=1/\mu L$. - $corr(\hat{r}_{s},\lambda_{n})\neq0.$ - $corr(\hat{r}_{e},\lambda_{n})\neq0.$ - $corr(I_{50}$,$d_{h})\neq0.$ - $corr(\omega_{8}$,$d_{h})\neq0$. - $corr(I_{50},\omega_{8})\neq0$. - $corr(\pi$,$\beta)\neq0$. - $corr(k_{t}$,$k_{h})\neq0.$ - $t_{sa}^{j}$ for ΔCCR5 and transplant groups was modeled as $\psi_{j}=\bar{\psi}e^{n_{j}+\varsigma_{t_{sa},\Delta CCR5}}$ and $\psi_{j}=\bar{\psi}e^{n_{j}+\varsigma_{t_{sa},\mathrm{WT}}}$, respectively. - $K_{p}^{j}$ for ΔCCR5 and transplant groups was modeled as $\psi_{j}={10}^{{\bar{\psi}+n}_{j}+\varsigma_{K_{p},\Delta CCR5}}$ and $\psi_{j}={10}^{{\bar{\psi}+n}_{j}+\varsigma_{K_{p},WT}}$ respectively. | 64.6 |
| 2 | - SHIV-specific CD8^+^ T cells kill SHIV-infected cells only ($\theta=0, \kappa>0$). - Immunity is lost during TBI: ($\omega_{8}$, $I_{50}$ and $d_{h}$ different during acute infection and after ATI). - SHIV-infection enhances activation of CD4^+^CCR5^-^ T cells leading to replenishment of CD4^+^CCR5^+^ T cells, and transient reduction of the CD4^+^CCR5^-^ compartment after ATI ($\omega_{4}>0$). - Does not include compartment *N_p2_* | - $\sigma_{t_{sa}}=1, \sigma_{\pi}=0.5$. - ${\psi_{j}^{ATI}=10}^{{\bar{\psi}+n}_{j}+\varsigma_{\psi,\mathrm{ATI}}}$ for $\omega_{8}$and $I_{50}$, and $\psi_{j}^{ATI}=\bar{\psi}e^{n_{j}+\varsigma_{\psi,\mathrm{ATI}}}$ for $d_{h}$. - $\kappa=1/\mu L$. - $corr(\hat{r}_{s},\lambda_{n})\neq0.$ - $corr(\hat{r}_{e},\lambda_{n})\neq0.$ - $corr(\hat{r}_{s},\beta)\neq0.$ - $corr(\hat{r}_{e},\beta)\neq0.$ - $corr(\hat{r}_{s},\pi)\neq0.$ - $corr(\hat{r}_{e},\pi)\neq0.$ - $corr(\beta,\lambda_{n})\neq0.$ - $corr(\pi,\lambda_{n})\neq0.$ - $corr(I_{50}$,$d_{h})\neq0.$ - $corr(\omega_{8}$,$d_{h})\neq0$. - $corr(I_{50},\omega_{8})\neq0$. - $corr(\pi$,$\beta)\neq0$. - $corr(k_{t}$,$k_{h})\neq0.$ - $t_{sa}^{j}$ for ΔCCR5 and transplant groups was modeled as $\psi_{j}=\bar{\psi}e^{n_{j}+\varsigma_{t_{sa},\Delta CCR5}}$ and $\psi_{j}=\bar{\psi}e^{n_{j}+\varsigma_{t_{sa},\mathrm{WT}}}$, respectively. - $K_{p}^{j}$ for ΔCCR5 and transplant groups was modeled as $\psi_{j}={10}^{{\bar{\psi}+n}_{j}+\varsigma_{K_{p},\Delta CCR5}}$ and $\psi_{j}={10}^{{\bar{\psi}+n}_{j}+\varsigma_{K_{p},WT}}$ respectively. | 1450.3 |
|  |  | - $\sigma_{t_{sa}}=1, \sigma_{\pi}=0.5$. - ${\psi_{j}^{ATI}=10}^{{\bar{\psi}+n}_{j}+\varsigma_{\psi,\mathrm{ATI}}}$ for $\omega_{8}$and $I_{50}$, and $\psi_{j}^{ATI}=\bar{\psi}e^{n_{j}+\varsigma_{\psi,\mathrm{ATI}}}$ for $d_{h}$. - $\kappa=1$ - $corr(\hat{r}_{s},\lambda_{n})\neq0.$ - $corr(\hat{r}_{e},\lambda_{n})\neq0.$ - $corr(I_{50}$,$d_{h})\neq0.$ - $corr(\omega_{8}$,$d_{h})\neq0$. - $corr(I_{50},\omega_{8})\neq0$. - $corr(\pi$,$\beta)\neq0$. - $corr(k_{t}$,$k_{h})\neq0.$ - $t_{sa}^{j}$ for ΔCCR5 and transplant groups was modeled as $\psi_{j}=\bar{\psi}e^{n_{j}+\varsigma_{t_{sa},\Delta CCR5}}$ and $\psi_{j}=\bar{\psi}e^{n_{j}+\varsigma_{t_{sa},\mathrm{WT}}}$, respectively. - $K_{p}^{j}$ for ΔCCR5 and transplant groups was modeled as $\psi_{j}={10}^{{\bar{\psi}+n}_{j}+\varsigma_{K_{p},\Delta CCR5}}$ and $\psi_{j}={10}^{{\bar{\psi}+n}_{j}+\varsigma_{K_{p},WT}}$ respectively. | 374.9 |
|  |  | - $\sigma_{t_{sa}}=1, \sigma_{\pi}=0.5$. - ${\psi_{j}^{ATI}=10}^{{\bar{\psi}+n}_{j}+\varsigma_{\psi,\mathrm{ATI}}}$ for $\omega_{8}$and $I_{50}$, and $\psi_{j}^{ATI}=\bar{\psi}e^{n_{j}+\varsigma_{\psi,\mathrm{ATI}}}$ for $d_{h}$. - $\kappa$ modeled as ${\psi_{j}=10}^{{\bar{\psi}+n}_{j}}$ - $corr(\hat{r}_{s},\lambda_{n})\neq0.$ - $corr(\hat{r}_{e},\lambda_{n})\neq0.$ - $corr(I_{50}$,$d_{h})\neq0.$ - $corr(\omega_{8}$,$d_{h})\neq0$. - $corr(I_{50},\omega_{8})\neq0$. - $corr(\pi$,$\beta)\neq0$. - $corr(k_{t}$,$k_{h})\neq0.$ - $t_{sa}^{j}$ for ΔCCR5 and transplant groups was modeled as $\psi_{j}=\bar{\psi}e^{n_{j}+\varsigma_{t_{sa},\Delta CCR5}}$ and $\psi_{j}=\bar{\psi}e^{n_{j}+\varsigma_{t_{sa},\mathrm{WT}}}$, respectively. - $K_{p}^{j}$ for ΔCCR5 and transplant groups was modeled as $\psi_{j}={10}^{{\bar{\psi}+n}_{j}+\varsigma_{K_{p},\Delta CCR5}}$ and $\psi_{j}={10}^{{\bar{\psi}+n}_{j}+\varsigma_{K_{p},WT}}$ respectively. | 255.6 |
| 3 | - SHIV-specific CD8^+^ T cells reduce virus production only ($\theta>0, \kappa=0$). - Immunity is *not* lost during TBI ($\omega_{8},I_{50},d_{h}$ equal during acute infection and after ATI). - SHIV-infection enhances activation of CD4^+^CCR5^-^ T cells leading to replenishment of CD4^+^CCR5^+^ T cells, and transient reduction of the CD4^+^CCR5^-^ compartment after ATI ($\omega_{4}>0$). - Does not include compartment *N_p2_* | - $\sigma_{t_{sa}}=1, \sigma_{\pi}=0.5$. - $\theta=1/\mu L$. - $corr(\hat{r}_{s},\lambda_{n})\neq0.$ - $corr(\hat{r}_{e},\lambda_{n})\neq0.$ - $corr(I_{50}$,$d_{h})\neq0.$ - $corr(\omega_{8}$,$d_{h})\neq0$. - $corr(I_{50},\omega_{8})\neq0$. - $corr(\pi$,$\beta)\neq0$. - $corr(k_{t}$,$k_{h})\neq0.$ - $t_{sa}^{j}$ for ΔCCR5 and transplant groups was modeled as $\psi_{j}=\bar{\psi}e^{n_{j}+\varsigma_{t_{sa},\Delta CCR5}}$ and $\psi_{j}=\bar{\psi}e^{n_{j}+\varsigma_{t_{sa},\mathrm{WT}}}$, respectively. - $K_{p}^{j}$ for ΔCCR5 and transplant groups was modeled as $\psi_{j}={10}^{{\bar{\psi}+n}_{j}+\varsigma_{K_{p},\Delta CCR5}}$ and $\psi_{j}={10}^{{\bar{\psi}+n}_{j}+\varsigma_{K_{p},WT}}$ respectively. | 709.2 |
| 4 | - SHIV-specific CD8^+^ T cells reduce virus production only ($\theta>0, \kappa=0$). - Immunity is lost during TBI: ($\omega_{8}$, $I_{50}$ and $d_{h}$ different during acute infection and after ATI). - SHIV-infection does not enhance activation of CD4^+^CCR5^-^ T cells or replenishment of CD4^+^CCR5^+^ T cells ($\omega_{4}=0$). - Does not include compartment *N_p2_* | - $\sigma_{t_{sa}}=1, \sigma_{\pi}=0.5$. - ${\psi_{j}^{ATI}=10}^{{\bar{\psi}+n}_{j}+\varsigma_{\psi,\mathrm{ATI}}}$ for $\omega_{8}$and $I_{50}$, and $\psi_{j}^{ATI}=\bar{\psi}e^{n_{j}+\varsigma_{\psi,\mathrm{ATI}}}$ for $d_{h}$. - $\theta=1/\mu L$. - $corr(\hat{r}_{s},\lambda_{n})\neq0.$ - $corr(\hat{r}_{e},\lambda_{n})\neq0.$ - $corr(I_{50}$,$d_{h})\neq0.$ - $corr(\omega_{8}$,$d_{h})\neq0$. - $corr(I_{50},\omega_{8})\neq0$. - $corr(\pi$,$\beta)\neq0$. - $corr(k_{t}$,$k_{h})\neq0.$ - $t_{sa}^{j}$ for ΔCCR5 and transplant groups was modeled as $\psi_{j}=\bar{\psi}e^{n_{j}+\varsigma_{t_{sa},\Delta CCR5}}$ and $\psi_{j}=\bar{\psi}e^{n_{j}+\varsigma_{t_{sa},\mathrm{WT}}}$, respectively. - $K_{p}^{j}$ for ΔCCR5 and transplant groups was modeled as $\psi_{j}={10}^{{\bar{\psi}+n}_{j}+\varsigma_{K_{p},\Delta CCR5}}$ and $\psi_{j}={10}^{{\bar{\psi}+n}_{j}+\varsigma_{K_{p},WT}}$ respectively. | 661.8 |
| 5 | - SHIV-specific CD8^+^ T cells reduce virus production only ($\theta>0, \kappa=0$). - Immunity is lost during TBI: ($\omega_{8}$, $I_{50}$ and $d_{h}$ different during acute infection and after ATI). - SHIV-infection enhances activation of CD4^+^CCR5^-^ T cells leading to replenishment of CD4^+^CCR5^+^ T cells, and transient reduction of the CD4^+^CCR5^-^ compartment after ATI ($\omega_{4}>0$). - Includes compartment *N_p2_* | - $\sigma_{t_{sa}}=1, \sigma_{\pi}=0.5$. - ${\psi_{j}^{ATI}=10}^{{\bar{\psi}+n}_{j}+\varsigma_{\psi,\mathrm{ATI}}}$ for $\omega_{8}$and $I_{50}$, and $\psi_{j}^{ATI}=\bar{\psi}e^{n_{j}+\varsigma_{\psi,\mathrm{ATI}}}$ for $d_{h}$. - $\theta=1/\mu L$. - $corr(I_{50}$,$d_{h})\neq0.$ - $corr(\omega_{8}$,$d_{h})\neq0$. - $corr(I_{50},\omega_{8})\neq0$. - $corr(\pi$,$\beta)\neq0$. | 77 |
|  |  | - $\sigma_{t_{sa}}=1, \sigma_{\pi}=0.5$. - ${\psi_{j}^{ATI}=10}^{{\bar{\psi}+n}_{j}+\varsigma_{\psi,\mathrm{ATI}}}$ for $\omega_{8}$and $I_{50}$, and $\psi_{j}^{ATI}=\bar{\psi}e^{n_{j}+\varsigma_{\psi,\mathrm{ATI}}}$ for $d_{h}$. - $\theta=1/\mu L$. - $corr(\hat{r}_{s},\lambda_{n})\neq0.$ - $corr(\hat{r}_{e},\lambda_{n})\neq0.$ - $corr(I_{50}$,$d_{h})\neq0.$ - $corr(\omega_{8}$,$d_{h})\neq0$. - $corr(I_{50},\omega_{8})\neq0$. - $corr(\pi$,$\beta)\neq0$. - $corr(k_{t}$,$k_{h})\neq0.$ - $corr(\pi$,$d_{h})\neq0$. - $corr(I_{50},\beta)\neq0$. - $corr(\pi$,$I_{50})\neq0$. - $corr(\omega_{8}$,$\beta)\neq0$. - $corr(\pi$,$\omega_{8})\neq0$. - $t_{sa}^{j}$ for ΔCCR5 and transplant groups was modeled as $\psi_{j}=\bar{\psi}e^{n_{j}+\varsigma_{t_{sa},\Delta CCR5}}$ and $\psi_{j}=\bar{\psi}e^{n_{j}+\varsigma_{t_{sa},\mathrm{WT}}}$, respectively. - $K_{p}^{j}$ for ΔCCR5 and transplant groups was modeled as $\psi_{j}={10}^{{\bar{\psi}+n}_{j}+\varsigma_{K_{p},\Delta CCR5}}$ and $\psi_{j}={10}^{{\bar{\psi}+n}_{j}+\varsigma_{K_{p},WT}}$ respectively. | 29.4 |
|  |  | - $\sigma_{t_{sa}}=1, \sigma_{\pi}=0.5$. - ${\psi_{j}^{ATI}=10}^{{\bar{\psi}+n}_{j}+\varsigma_{\psi,\mathrm{ATI}}}$ for $\omega_{8}$and $I_{50}$, and $\psi_{j}^{ATI}=\bar{\psi}e^{n_{j}+\varsigma_{\psi,\mathrm{ATI}}}$ for $d_{h}$. - $\theta=1/\mu L$. - $corr(\hat{r}_{s},\lambda_{n})\neq0.$ - $corr(\hat{r}_{e},\lambda_{n})\neq0.$ - $corr(\hat{r}_{s},\beta)\neq0.$ - $corr(\hat{r}_{e},\beta)\neq0.$ - $corr(\hat{r}_{s},\pi)\neq0.$ - $corr(\hat{r}_{e},\pi)\neq0.$ - $corr(\beta,\lambda_{n})\neq0.$ - $corr(\pi,\lambda_{n})\neq0.$ - $corr(I_{50}$,$d_{h})\neq0.$ - $corr(\omega_{8}$,$d_{h})\neq0$. - $corr(I_{50},\omega_{8})\neq0$. - $corr(\pi$,$\beta)\neq0$. - $corr(k_{t}$,$k_{h})\neq0.$ - $t_{sa}^{j}$ for ΔCCR5 and transplant groups was modeled as $\psi_{j}=\bar{\psi}e^{n_{j}+\varsigma_{t_{sa},\Delta CCR5}}$ and $\psi_{j}=\bar{\psi}e^{n_{j}+\varsigma_{t_{sa},\mathrm{WT}}}$, respectively. - $K_{p}^{j}$ for ΔCCR5 and transplant groups was modeled as $\psi_{j}={10}^{{\bar{\psi}+n}_{j}+\varsigma_{K_{p},\Delta CCR5}}$ and $\psi_{j}={10}^{{\bar{\psi}+n}_{j}+\varsigma_{K_{p},WT}}$ respectively. | 69.3 |
|  |  | - $\sigma_{t_{sa}}=1, \sigma_{\pi}=0.5$. - ${\psi_{j}^{ATI}=10}^{{\bar{\psi}+n}_{j}+\varsigma_{\psi,\mathrm{ATI}}}$ for $\omega_{8}$and $I_{50}$, and $\psi_{j}^{ATI}=\bar{\psi}e^{n_{j}+\varsigma_{\psi,\mathrm{ATI}}}$ for $d_{h}$. - $\theta=1/\mu L$. - $corr(\hat{r}_{s},\lambda_{n})\neq0.$ - $corr(\hat{r}_{e},\lambda_{n})\neq0.$ - $corr(I_{50}$,$d_{h})\neq0.$ - $corr(\omega_{8}$,$d_{h})\neq0$. - $corr(I_{50},\omega_{8})\neq0$. - $corr(\pi$,$\beta)\neq0$. - $corr(k_{t}$,$k_{h})\neq0.$ - $t_{sa}^{j}$ for ΔCCR5 and transplant groups was modeled as $\psi_{j}=\bar{\psi}e^{n_{j}+\varsigma_{t_{sa},\Delta CCR5}}$ and $\psi_{j}=\bar{\psi}e^{n_{j}+\varsigma_{t_{sa},\mathrm{WT}}}$, respectively. - $K_{p}^{j}$ for ΔCCR5 and transplant groups was modeled as $\psi_{j}={10}^{{\bar{\psi}+n}_{j}+\varsigma_{K_{p},\Delta CCR5}}$ and $\psi_{j}={10}^{{\bar{\psi}+n}_{j}+\varsigma_{K_{p},WT}}$ respectively. | **0** |
| 6 | - SHIV-specific CD8^+^ T cells kill SHIV-infected cells only ($\theta=0, \kappa>0$). - Immunity is lost during TBI: ($\omega_{8}$, $I_{50}$ and $d_{h}$ different during acute infection and after ATI). - SHIV-infection enhances activation of CD4^+^CCR5^-^ T cells leading to replenishment of CD4^+^CCR5^+^ T cells, and transient reduction of the CD4^+^CCR5^-^ compartment after ATI ($\omega_{4}>0$). - Includes compartment *N_p2_* | - $\sigma_{t_{sa}}=1, \sigma_{\pi}=0.5$. - ${\psi_{j}^{ATI}=10}^{{\bar{\psi}+n}_{j}+\varsigma_{\psi,\mathrm{ATI}}}$ for $\omega_{8}$and $I_{50}$, and $\psi_{j}^{ATI}=\bar{\psi}e^{n_{j}+\varsigma_{\psi,\mathrm{ATI}}}$ for $d_{h}$. - $\kappa=1/\mu L$. - $corr(\hat{r}_{s},\lambda_{n})\neq0.$ - $corr(\hat{r}_{e},\lambda_{n})\neq0.$ - $corr(\hat{r}_{s},\beta)\neq0.$ - $corr(\hat{r}_{e},\beta)\neq0.$ - $corr(\hat{r}_{s},\pi)\neq0.$ - $corr(\hat{r}_{e},\pi)\neq0.$ - $corr(\beta,\lambda_{n})\neq0.$ - $corr(\pi,\lambda_{n})\neq0.$ - $corr(I_{50}$,$d_{h})\neq0.$ - $corr(\omega_{8}$,$d_{h})\neq0$. - $corr(I_{50},\omega_{8})\neq0$. - $corr(\pi$,$\beta)\neq0$. - $corr(k_{t}$,$k_{h})\neq0.$ - $t_{sa}^{j}$ for ΔCCR5 and transplant groups was modeled as $\psi_{j}=\bar{\psi}e^{n_{j}+\varsigma_{t_{sa},\Delta CCR5}}$ and $\psi_{j}=\bar{\psi}e^{n_{j}+\varsigma_{t_{sa},\mathrm{WT}}}$, respectively. - $K_{p}^{j}$ for ΔCCR5 and transplant groups was modeled as $\psi_{j}={10}^{{\bar{\psi}+n}_{j}+\varsigma_{K_{p},\Delta CCR5}}$ and $\psi_{j}={10}^{{\bar{\psi}+n}_{j}+\varsigma_{K_{p},WT}}$ respectively. | 1987.4 |
|  |  | - $\sigma_{t_{sa}}=1, \sigma_{\pi}=0.5$. - ${\psi_{j}^{ATI}=10}^{{\bar{\psi}+n}_{j}+\varsigma_{\psi,\mathrm{ATI}}}$ for $\omega_{8}$and $I_{50}$, and $\psi_{j}^{ATI}=\bar{\psi}e^{n_{j}+\varsigma_{\psi,\mathrm{ATI}}}$ for $d_{h}$. - $\kappa=1$ - $corr(\hat{r}_{s},\lambda_{n})\neq0.$ - $corr(\hat{r}_{e},\lambda_{n})\neq0.$ - $corr(I_{50}$,$d_{h})\neq0.$ - $corr(\omega_{8}$,$d_{h})\neq0$. - $corr(I_{50},\omega_{8})\neq0$. - $corr(\pi$,$\beta)\neq0$. - $corr(k_{t}$,$k_{h})\neq0.$ - $t_{sa}^{j}$ for ΔCCR5 and transplant groups was modeled as $\psi_{j}=\bar{\psi}e^{n_{j}+\varsigma_{t_{sa},\Delta CCR5}}$ and $\psi_{j}=\bar{\psi}e^{n_{j}+\varsigma_{t_{sa},\mathrm{WT}}}$, respectively. - $K_{p}^{j}$ for ΔCCR5 and transplant groups was modeled as $\psi_{j}={10}^{{\bar{\psi}+n}_{j}+\varsigma_{K_{p},\Delta CCR5}}$ and $\psi_{j}={10}^{{\bar{\psi}+n}_{j}+\varsigma_{K_{p},WT}}$ respectively. | 317.6 |
|  |  | - $\sigma_{t_{sa}}=1, \sigma_{\pi}=0.5$. - ${\psi_{j}^{ATI}=10}^{{\bar{\psi}+n}_{j}+\varsigma_{\psi,\mathrm{ATI}}}$ for $\omega_{8}$and $I_{50}$, and $\psi_{j}^{ATI}=\bar{\psi}e^{n_{j}+\varsigma_{\psi,\mathrm{ATI}}}$ for $d_{h}$. - $\kappa$ modeled as ${\psi_{j}=10}^{{\bar{\psi}+n}_{j}}$ - $corr(\hat{r}_{s},\lambda_{n})\neq0.$ - $corr(\hat{r}_{e},\lambda_{n})\neq0.$ - $corr(I_{50}$,$d_{h})\neq0.$ - $corr(\omega_{8}$,$d_{h})\neq0$. - $corr(I_{50},\omega_{8})\neq0$. - $corr(\pi$,$\beta)\neq0$. - $corr(k_{t}$,$k_{h})\neq0.$ - $t_{sa}^{j}$ for ΔCCR5 and transplant groups was modeled as $\psi_{j}=\bar{\psi}e^{n_{j}+\varsigma_{t_{sa},\Delta CCR5}}$ and $\psi_{j}=\bar{\psi}e^{n_{j}+\varsigma_{t_{sa},\mathrm{WT}}}$, respectively. - $K_{p}^{j}$ for ΔCCR5 and transplant groups was modeled as $\psi_{j}={10}^{{\bar{\psi}+n}_{j}+\varsigma_{K_{p},\Delta CCR5}}$ and $\psi_{j}={10}^{{\bar{\psi}+n}_{j}+\varsigma_{K_{p},WT}}$ respectively. | 380.9 |
