# Supplementary material for: Thresholds for post-rebound SHIV control after CCR5 gene-edited autologous hematopoietic cell transplantation
Source: eLife. 2021 Jan 12;10:e57646. doi: 10.7554/eLife.57646 (PMC7803377; doi:10.7554/eLife.57646)
Supplement: Figure 5—source data 2. — RSE: relative standard error. Empty fields represent cases when the standard deviation of random effects, σψ, was fixed to zero. Values of ψ¯ for β,ω4,ω8, and I50 shown here are transformed assuming a blood volume of 3 × 105 μL (calculated assuming blood:weight ratio of 60 mL/kg and body weight of 5 kg). Red values represent an RSE greater than 100% implying that the number of data points may not be enough to estimate the respective parameter. [file elife-57646-fig5-data2.docx]

**Figure 5-source data 2.** Population parameter estimates for the fits of the model with lowest AIC in **Figure 5-source data 1** to the T cell and virus dynamics. RSE: relative standard error. Empty fields represent cases when the standard deviation of random effects, $\boldsymbol{\sigma}_{\boldsymbol{\psi}}$ , was fixed to zero. Values of $\bar{\psi}$ for $\beta,\omega_{4},\omega_{8}$ and $I_{50}$ shown here are transformed assuming a blood volume of 3×10^5^ μL (calculated assuming blood:weight ratio of 60mL/Kg and body weight of 5Kg). Red values represent an RSE greater than 100% implying that the number of data points may not be enough to estimate the respective parameter.

| **Parameter** | $\bar{\boldsymbol{\psi}}$ | $\boldsymbol{\sigma}_{\boldsymbol{\psi}}$ | **%RSE for:** | |
| --- | --- | --- | --- | --- |
|  |  |  | $\bar{\boldsymbol{\psi}}$ | $\boldsymbol{\sigma}_{\boldsymbol{\psi}}$ |
| $\boldsymbol{K}_{\boldsymbol{p}}$ | 3.2 | 0.2 | 1 |  |
|  | $\varsigma_{K_{p},WT}=-0.02$ |  | 500 |  |
|  | $\varsigma_{K_{p},\Delta CCR5}=0.02$ |  | 223 |  |
| $\boldsymbol{\beta}$ | -3.9 | 0.4 | 38 | 15 |
| $\boldsymbol{\pi}$ | 5.3 | 0.5 | 2 |  |
| $\boldsymbol{\omega}_{\boldsymbol{4}}$ | -2.2 | 0.4 | 1 | 22 |
| $\boldsymbol{\omega}_{\boldsymbol{8}}$ | -2.9 | 0.7 | 1.8 | 19 |
|  | $\varsigma_{\omega_{8},\mathrm{ATI}}=1.21$ | 0.8 | 21 | 23 |
| $\boldsymbol{I}_{\mathbf{50}}$ | 0.2 | 0.7 | 3 | 22 |
|  | $\varsigma_{I_{50},\mathrm{ATI}}=0.85$ | 0.6 | 29 | 34 |
| $\boldsymbol{d}_{\boldsymbol{h}}$ | 0.005 | 0.6 | 15 | 25 |
|  | $\varsigma_{d_{h},\mathrm{ATI}}=2.7$ | 1.9 | 20 | 20 |
| $\boldsymbol{t}_{\boldsymbol{sa}}$ | 4.7 | 1 | 40 |  |
|  | $\varsigma_{t_{sa},WT}=1.0$ |  | 62 |  |
|  | $\varsigma_{t_{sa},\Delta CCR5}=1.5$ |  | 30 |  |
| $\boldsymbol{k}_{\boldsymbol{T}}$ | 0.5 | 0.1 | 4 | 21 |
| $\boldsymbol{k}_{\boldsymbol{H}}$ | 1.14 | 0.1 | 4 | 21 |
|  | **Parameter value** | | **%RSE** | |
| $\boldsymbol{corr(}{\hat{\boldsymbol{r}}}_{\boldsymbol{s}}\boldsymbol{,}\boldsymbol{\lambda}_{\boldsymbol{n}}\boldsymbol{)}$ | 0.6 | | 22 | |
| $\boldsymbol{corr(}{\hat{\boldsymbol{r}}}_{\boldsymbol{e}}\boldsymbol{,}\boldsymbol{\lambda}_{\boldsymbol{n}}\boldsymbol{)}$ | 0.6 | | 25 | |
| $\boldsymbol{corr(}\boldsymbol{I}_{\boldsymbol{50}}$**,**$\boldsymbol{d}_{\boldsymbol{h}}\boldsymbol{)}$ | -0.96 | | 16 | |
| $\boldsymbol{corr}\boldsymbol{(}\boldsymbol{\omega}_{\boldsymbol{8}}$**,**$\boldsymbol{d}_{\boldsymbol{h}}\boldsymbol{)}$ | 0.55 | | 41 | |
| $\boldsymbol{corr}\boldsymbol{(}\boldsymbol{I}_{\boldsymbol{50}}\mathbf{,}\boldsymbol{\omega}_{\boldsymbol{8}}\boldsymbol{)}$ | -0.77 | | 14 | |
| $\boldsymbol{corr}\boldsymbol{(}\boldsymbol{\pi}$**,**$\boldsymbol{\beta}\boldsymbol{)}$ | -0.82 | | 8 | |
| $\boldsymbol{corr}\boldsymbol{(}\boldsymbol{k}_{\boldsymbol{T}}$**,**$\boldsymbol{k}_{\boldsymbol{H}}\boldsymbol{)}$ | -0.92 | | 6 | |
| $\boldsymbol{\sigma}_{\boldsymbol{N}}$ | 0.2 | | 2 | |
| $\boldsymbol{\sigma}_{\boldsymbol{R}}$ | 0.16 | | 2 | |
| $\boldsymbol{\sigma}_{\boldsymbol{C}_{\boldsymbol{4}}}$ | 0.14 | | 1.8 | |
| $\boldsymbol{\sigma}_{\boldsymbol{C}_{\boldsymbol{8}}}$ | 0.19 | | 1.8 | |
| $\boldsymbol{\sigma}_{\boldsymbol{V}}$ | 0.5 | | 2.6 | |
| $\boldsymbol{\sigma}_{\boldsymbol{E}}$ | 0.18 | | 9.4 | |
| $\boldsymbol{\sigma}_{\boldsymbol{M}}$ | 0.3 | | 9.6 | |
