# Supplementary material for: Thresholds for post-rebound SHIV control after CCR5 gene-edited autologous hematopoietic cell transplantation
Source: eLife. 2021 Jan 12;10:e57646. doi: 10.7554/eLife.57646 (PMC7803377; doi:10.7554/eLife.57646)
Supplement: Figure 5—source data 3. — Values of ψ¯ for β,ω4,ω8, and I50 shown here are transformed assuming a blood volume of 3 × 105 μL (calculated assuming blood:weight ratio of 60 mL/kg and body weight of 5 kg). Shown are individual estimates for animals that continued study after ATI. [file elife-57646-fig5-data3.docx]

**Figure 5-source data 3.** Individual parameter estimates for the fits of the model in **equations 2-3** in main text (lowest AIC in **Figure 5-source data 1**) to the T cell and virus dynamics. Values of $\bar{\psi}$ for $\beta,\omega_{4},\omega_{8}$ and $I_{50}$ shown here are transformed assuming a blood volume of 3×10^5^ μL (calculated assuming blood:weight ratio of 60mL/Kg and body weight of 5Kg). Shown are individual estimates for animals that continued study after ATI.

|  | **Control** | | | | **WT-Transplant** | | | | **ΔCCR5-Transplant** | | | | | |
| --- | --- | --- | --- | --- | --- | --- | --- | --- | --- | --- | --- | --- | --- | --- |
| **Par.**  **ID** | **Z09087** | **Z09106** | **Z09192** | **Z09204** | **Z09144** | **Z08214** | **A11200** | **Z09196** | **A11219** | **T10187** | **R10159** | **T10173** | **Z11151** | **Z12420** |
| ${\hat{\boldsymbol{r}}}_{\boldsymbol{p}}^{\boldsymbol{j}}$  **(1/day)** | 0.01 | 0.03 | 0.04 | 0.04 | 0.03 | 0.02 | 0.02 | 0.06 | 0.01 | 0.04 | 0.03 | 0.02 | 0.03 | 0.06 |
| ${\hat{\boldsymbol{r}}}_{\boldsymbol{s}}^{\boldsymbol{j}}$  **(1/day)** | 0.05 | 0.06 | 0.08 | 0.06 | 0.09 | 0.12 | 0.07 | 0.14 | 0.11 | 0.10 | 0.07 | 0.06 | 0.06 | 0.08 |
| ${\hat{\boldsymbol{r}}}_{\boldsymbol{m}}^{\boldsymbol{j}}$ **(1/day)** | 0.03 | 0.04 | 0.05 | 0.03 | 0.06 | 0.08 | 0.05 | 0.13 | 0.05 | 0.06 | 0.05 | 0.04 | 0.02 | 0.07 |
| ${\hat{\boldsymbol{r}}}_{\boldsymbol{e}}^{\boldsymbol{j}}$  **(1/day)** | 0.03 | 0.03 | 0.03 | 0.03 | 0.03 | 0.03 | 0.03 | 0.03 | 0.03 | 0.03 | 0.03 | 0.03 | 0.03 | 0.03 |
| ${\hat{\boldsymbol{d}}}_{\boldsymbol{n}}^{\boldsymbol{j}}$  **(1/day)** | 0.01 | 0.03 | 0.03 | 0.02 | 0.02 | 0.02 | 0.02 | 0.03 | 0.04 | 0.03 | 0.04 | 0.04 | 0.02 | 0.03 |
| $\boldsymbol{\lambda}_{\boldsymbol{p}}^{\boldsymbol{j}}$  **(1/day)** | 0.004 | 0.004 | 0.004 | 0.004 | 0.004 | 0.004 | 0.004 | 0.004 | 0.004 | 0.004 | 0.004 | 0.004 | 0.004 | 0.004 |
| $\boldsymbol{\lambda}_{\boldsymbol{n}}^{\boldsymbol{j}}$  **(1/day)** | 0.002 | 0.004 | 0.004 | 0.003 | 0.003 | 0.004 | 0.003 | 0.005 | 0.006 | 0.005 | 0.006 | 0.006 | 0.003 | 0.005 |
| $\boldsymbol{\lambda}_{\boldsymbol{s}}^{\boldsymbol{j}}$  **(1/day)** | 0.02 | 0.01 | 0.01 | 0.01 | 0.04 | 0.01 | 0.01 | 0.03 | 0.02 | 0.01 | 0.02 | 0.01 | 0.03 | 0.01 |
| $\boldsymbol{\lambda}_{\boldsymbol{m}}^{\boldsymbol{j}}$ **(1/day)** | 0.08 | 0.08 | 0.08 | 0.08 | 0.08 | 0.08 | 0.08 | 0.08 | 0.08 | 0.08 | 0.08 | 0.08 | 0.08 | 0.08 |
| $\boldsymbol{K}_{\boldsymbol{p}}^{\boldsymbol{j}}$  $\left( \boldsymbol{cells} \right)$ | 10^8.8^ | 10^8.7^ | 10^8.9^ | 10^8.9^ | 10^8.6^ | 10^8.8^ | 10^8.7^ | 10^8.6^ | 10^8.5^ | 10^8.6^ | 10^8.9^ | 10^8.7^ | 10^8.8^ | 10^8.6^ |
| $\boldsymbol{K}_{\boldsymbol{s}}^{\boldsymbol{j}}$  $\left( \frac{\boldsymbol{cells}}{\boldsymbol{\mu L}} \right)$ | 1451.7 | 1203.6 | 1919.4 | 1596.1 | 875.6 | 1361.1 | 1008.3 | 789.9 | 717.1 | 959.9 | 1862.2 | 1175.3 | 1478.9 | 843.6 |
| $\boldsymbol{K}_{\boldsymbol{m}}^{\boldsymbol{j}}$  $\left( \frac{\boldsymbol{cells}}{\boldsymbol{\mu L}} \right)$ | 543.4 | 687.4 | 813.0 | 432.7 | 271.6 | 413.4 | 369.5 | 350.0 | 163.4 | 248.6 | 407.9 | 206.5 | 419.3 | 256.9 |
| $\boldsymbol{K}_{\boldsymbol{e}}^{\boldsymbol{j}}$  $\left( \frac{\boldsymbol{cells}}{\boldsymbol{\mu L}} \right)$ | 1314.2 | 1089.6 | 1737.7 | 1445.0 | 792.7 | 1232.2 | 912.8 | 715.1 | 649.2 | 869.0 | 1685.9 | 1064.0 | 1338.9 | 763.8 |
| $\boldsymbol{k}_{\boldsymbol{T}}^{\boldsymbol{j}}$  **(1/day)** | 0.54 | 0.55 | 0.54 | 0.54 | 0.56 | 0.70 | 0.45 | 0.57 | 0.55 | 0.46 | 0.55 | 0.43 | 0.56 | 0.58 |
| $\boldsymbol{k}_{\boldsymbol{H}}^{\boldsymbol{j}}$  **(1/day)** | 1.14 | 1.13 | 1.13 | 1.14 | 1.10 | 1.18 | 1.09 | 1.13 | 1.13 | 1.16 | 1.25 | 1.18 | 1.13 | 1.12 |
| $\boldsymbol{\beta}^{\boldsymbol{j}}$ $\left( \frac{\boldsymbol{\mu L}}{\boldsymbol{copies*day}} \right)$ | 0.0009 | 0.0004 | 0.0001 | 0.0004 | 0.0001 | 0.0001 | 0.0001 | 0.0002 | 0.0001 | 0.0002 | 0.0000 | 0.0001 | 0.0011 | 0.0002 |
| $\boldsymbol{t}_{\boldsymbol{sa}}^{\boldsymbol{j}}$ **(days)** | 3.1 | 3.8 | 8.4 | 4.2 | 9.6 | 16.1 | 11.1 | 14.0 | 17.3 | 8.2 | 10.3 | 15.8 | 12.3 | 46.0 |
| $\boldsymbol{\pi}^{\boldsymbol{j}}$  **(1/day)** | 10^5.0^ | 10^5.6^ | 10^5.2^ | 10^4.7^ | 10^5.7^ | 10^5.9^ | 10^5.4^ | 10^5.5^ | 10^5.4^ | 10^5.2^ | 10^5.4^ | 10^5.3^ | 10^4.4^ | 10^5.1^ |
| $\boldsymbol{\omega}_{\boldsymbol{4}}^{\boldsymbol{j}}$ $\left( \frac{\boldsymbol{\mu L}}{\boldsymbol{cells*day}} \right)$ | 0.004 | 0.011 | 0.002 | 0.007 | 0.016 | 0.005 | 0.003 | 0.010 | 0.003 | 0.068 | 0.003 | 0.009 | 0.008 | 0.007 |
| $\boldsymbol{\omega}_{\boldsymbol{8}}^{\boldsymbol{j}}$ $\left( \frac{\boldsymbol{\mu L}}{\boldsymbol{cells*day}} \right)$ | 0.0002 | 0.0016 | 0.0006 | 0.0136 | 0.0029 | 0.0088 | 0.0119 | 0.0015 | 0.0007 | 0.0014 | 0.0003 | 0.0014 | 0.0002 | 0.0109 |
| $\boldsymbol{\omega}_{\boldsymbol{8}}^{\boldsymbol{j,ATI}}$ $\left( \frac{\boldsymbol{\mu L}}{\boldsymbol{cells*day}} \right)$ | 0.0006 | 0.004 | 0.0009 | 0.73 | 0.27 | 0.01 | 0.0336 | 0.02 | 0.03 | 0.0006 | 0.02 | 0.21 | 0.009 | 0.34 |
| $\boldsymbol{d}_{\boldsymbol{h}}^{\boldsymbol{j}}$  **(1/day)** | 0.005 | 0.004 | 0.004 | 0.009 | 0.009 | 0.009 | 0.008 | 0.005 | 0.004 | 0.008 | 0.003 | 0.003 | 0.005 | 0.008 |
| $\boldsymbol{d}_{\boldsymbol{h}}^{\boldsymbol{j,ATI}}$  $\mathbf{(1/day)}$ | 0.005 | 0.008 | 0.023 | 0.339 | 1.162 | 0.827 | 0.020 | 0.213 | 0.240 | 0.021 | 0.022 | 0.047 | 0.377 | 0.041 |
| $\boldsymbol{I}_{\mathbf{50}}^{\boldsymbol{j}}$  $\left( \frac{\boldsymbol{cells}}{\boldsymbol{\mu L}} \right)$ | 2.7 | 1.8 | 2.8 | 0.2 | 0.3 | 0.2 | 0.3 | 1.2 | 3.4 | 0.6 | 9.1 | 3.9 | 3.5 | 0.3 |
| $\boldsymbol{I}_{\mathbf{50}}^{\boldsymbol{j,ATI}}$  $\left( \frac{\boldsymbol{cells}}{\boldsymbol{\mu L}} \right)$ | 9.2 | 3.4 | 10.2 | 1.3 | 1.5 | 16.4 | 7.5 | 6.7 | 68.4 | 3.4 | 18.5 | 32.4 | 61.1 | 2.0 |
