# Supplementary material for: Thresholds for post-rebound SHIV control after CCR5 gene-edited autologous hematopoietic cell transplantation
Source: eLife. 2021 Jan 12;10:e57646. doi: 10.7554/eLife.57646 (PMC7803377; doi:10.7554/eLife.57646)
Supplement: Transparent reporting form [file elife-57646-transrepform.docx]

***eLife’s* transparent reporting form**

We encourage authors to provide detailed information *within their submission* to facilitate the interpretation and replication of experiments. Authors can upload supporting documentation to indicate the use of appropriate reporting guidelines for health-related research (see [EQUATOR Network](http://www.equator-network.org/%20)), life science research (see the [BioSharing Information Resource](https://biosharing.org/)), or the [ARRIVE guidelines](http://www.plosbiology.org/article/info:doi/10.1371/journal.pbio.1000412) for reporting work involving animal research. Where applicable, authors should refer to any relevant reporting standards documents in this form.

If you have any questions, please consult our Journal Policies and/or contact us: [editorial@elifesciences.org](mailto:editorial@elifesciences.org).

**Sample-size estimation**

- You should state whether an appropriate sample size was computed when the study was being designed
- You should state the statistical method of sample size computation and any required assumptions
- If no explicit power analysis was used, you should describe how you decided what sample (replicate) size (number) to use

Please outline where this information can be found within the submission (e.g., sections or figure legends), or explain why this information doesn’t apply to your submission:

In this study we developed mathematical models to analyze data published before, <https://doi.org/10.1371/journal.ppat.1006956>. Sample-size estimations are addressed in that publication.

**Replicates**

- You should report how often each experiment was performed
- You should include a definition of biological versus technical replication
- The data obtained should be provided and sufficient information should be provided to indicate the number of independent biological and/or technical replicates
- If you encountered any outliers, you should describe how these were handled
- Criteria for exclusion/inclusion of data should be clearly stated
- High-throughput sequence data should be uploaded before submission, with a private link for reviewers provided (these are available from both GEO and ArrayExpress)

Please outline where this information can be found within the submission (e.g., sections or figure legends), or explain why this information doesn’t apply to your submission:

In this study we developed a mathematical model to analyze data published before in <https://doi.org/10.1371/journal.ppat.1006956>. A brief summary of the study is presented in the methods sections. All other experimental details are in the previous publication.

**Statistical reporting**

- Statistical analysis methods should be described and justified
- Raw data should be presented in figures whenever informative to do so (typically when N per group is less than 10)
- For each experiment, you should identify the statistical tests used, exact values of N, definitions of center, methods of multiple test correction, and dispersion and precision measures (e.g., mean, median, SD, SEM, confidence intervals; and, for the major substantive results, a measure of effect size (e.g., Pearson's r, Cohen's d)
- Report exact p-values wherever possible alongside the summary statistics and 95% confidence intervals. These should be reported for all key questions and not only when the p-value is less than 0.05.

Please outline where this information can be found within the submission (e.g., sections or figure legends), or explain why this information doesn’t apply to your submission:

- The main statistical analysis for data fitting was done using nonlinear mixed effects modeling and is described in detail in the Methods section.

- Statistical Akaike Information Criteria for model selection is described in the Methods section.

- Non-parametric, correlation tests and p-values are described in each figure legend.

(For large datasets, or papers with a very large number of statistical tests, you may upload a single table file with tests, Ns, etc., with reference to sections in the manuscript.)

**Group allocation**

- Indicate how samples were allocated into experimental groups (in the case of clinical studies, please specify allocation to treatment method); if randomization was used, please also state if restricted randomization was applied
- Indicate if masking was used during group allocation, data collection and/or data analysis

Please outline where this information can be found within the submission (e.g., sections or figure legends), or explain why this information doesn’t apply to your submission:

Each experimental group is described in the Methods section with the schematic in Fig 1. Details of how these groups were selected can be found in: <https://doi.org/10.1371/journal.ppat.1006956>.

**Additional data files (“source data”)**

- We encourage you to upload relevant additional data files, such as numerical data that are represented as a graph in a figure, or as a summary table
- Where provided, these should be in the most useful format, and they can be uploaded as “Source data” files linked to a main figure or table
- Include model definition files including the full list of parameters used
- Include code used for data analysis (e.g., R, MatLab)
- Avoid stating that data files are “available upon request”

Please indicate the figures or tables for which source data files have been provided:

**Figure 2-source data.** Complete data set of blood T cell counts for **Figures 2** and **3**.

**Figure 2-source code.** R code for plots and tests in Figure 2.

**Figure-3-source code 1.** Best model file for T cell reconstitution in Monolix format.

**Figure 3-source data 1.** Values of the fraction of protected cells in transplant product $f_{p}$, dose or number of HSPCs in transplant product $D$ and time of transplantation $t_{x}$ of each animal for model fitting and projections. We assumed animal weight of 5Kg.

**Figure 3-source data 2.** Competing models for fitting T cell reconstitution with respective AIC values. Best fit in bold-red (lowest AIC). The AIC values presented for each statistical assumption is the lowest of 10 runs of the SAEM algorithm with different randomly selected initial guesses.

**Figure 3-source data 3.** Population parameter estimates for the best fits of the model in **equation 2** in the main text (lowest AIC in **Figure 3- source data 2**) to the T cell reconstitution dynamics.

**Figure 3-source data 4.** Individual parameter estimates for the best fits of the model in **equation 2** in the main text (lowest AIC in **Figure 3-source data 2**) to the T cell reconstitution dynamics.

**Figure 3-source data 5.** Population parameter estimates for the best fits used in the R code for Figure 3.

**Figure 3-source code 2.** R code for plots in Figure 3.

**Figure 4-source data.** Complete data set of blood T cell counts and viral load for Figures 4 and 5.

**Figure 4-source code.** R code for plots and test in Figure 4.

**Figure-5-source code 1.** Best model file for T cell and virus dynamics from acute infection after ATI in Monolix format.

**Figure 5-source data 1.** Competing models for fitting T cell and viral dynamics (**equations 2-3** in main text) using the best model in **Figure 3-source data 2** and fixing parameter values as in **Figure 3-source data 3**, with AIC values.

**Figure 5-source data 2.** Population parameter estimates for the fits of the model with lowest AIC in **Figure 5-source data 1** to the T cell and virus dynamics.

**Figure 5-source data 3.** Individual parameter estimates for the fits of the model in **equations 2-3** in main text (lowest AIC in **Figure 5-source data 1**) to the T cell and virus dynamics.

**Figure 5-source data 4.** Individual parameter estimates obtained from Monolix for the best fits used in the R code for Figure 5.

**Figure 5-source code 2.** R code for plots in Figure 5B.

**Figure 5-source code 3.** R code for plots and tests in Figure 5C-D.

**Figure 6-source data.** Results from all simulations varying *f_p_*, *D* and *P_r_*.

**Figure 6-source code 1.** R code for plots in Figure 6A-B.

**Figure 6-source code 2.** R code for plots in Figure 6C-D.

**Figure 7-source data.** Results from all simulations varying time to ATI.
